# Data of bromide sorption experiments with woodchips and tracer testing of denitrification beds

**DOI:** 10.1016/j.dib.2019.103914

**Published:** 2019-04-17

**Authors:** Ehsan Ghane, Gary W. Feyereisen, Carl J. Rosen

**Affiliations:** a Department of Biosystems and Agricultural Engineering, Michigan State University, East Lansing, MI 48824, USA; b Soil and Water Management Research Unit, USDA Agricultural Research Service, Saint Paul, MN 55108, USA; c Department of Soil, Water, and Climate, University of Minnesota, Saint Paul, MN 55108, USA

**Keywords:** Denitrifying bioreactor, Effective porosity, Hydraulic retention time, Subsurface drainage, Tile drainage, Woodchip bioreactor

## Abstract

Three different woodchip forms were tested for bromide sorption including ground woodchip, unwashed woodchips, and washed woodchips. We used six varying initial bromide concentrations to conduct the bromide sorption experiments with each woodchip form. Data on the initial and equilibrium bromide concentrations, wood mass, and initial and equilibrium solution pH from each of the six experiments are presented. Seven bromide tracer tests were conducted on field-scale denitrification beds. In this paper, data from each of the tracer tests including variation of bromide concentration over time and hydraulic indices of the tracer tests are presented. Interpretation of the data can be found in the research article entitled “Efficacy of bromide tracers for evaluating the hydraulic performance of denitrification beds treating agricultural drainage water” [1].

**Table:** Specifications Table

Subject area	*Agriculture, ecosystem*
More specific subject area	*Edge-of-field conservation practice*
Type of data	*Table, image, figure*
How data was acquired	*In the laboratory experiments, bromide was determined by Ion Chromatography (Thermo Scientific, Dionex Integrion HPIC, San Jose, CA, USA). In the field experiments, bromide was determined by colorimetry (Lachat QuikChem 8500 Flow Injection Analysis, Hach Co., Loveland, CO, USA)*
Data format	*Raw and analyzed*
Experimental factors	*For laboratory sorption experiment 1, air-dried woodchips were ground into particle size of <1 mm. For sorption experiments 2 and 3, unwashed and washed woodchips were used, respectively. For the laboratory experiments, we prepared six initial bromide concentrations ranging from 6.1 to 69.9 mg L*^*−1*^*. For the field bromide tracer experiments, denitrification bed numbers 2 to 8 were used.*
Experimental features	*Laboratory sorption experiment and field tracer testing*
Data source location	*Willmar, USA*
Data accessibility	*Data are in this article*
Related research article	E. Ghane, G.W. Feyereisen, C.J. Rosen. Efficacy of bromide tracers for evaluating the hydraulic performance of denitrification beds treating agricultural drainage water. Journal of Hydrology. https://doi.org/10.1016/j.jhydrol.2019.02.031[1]

**Table:** 

**Value of the Data** • These data can be used in the development of further bromide sorption experiments • These data can help researchers gain a better understanding of how bromide tracers move in denitrification beds • These data provide a guide on conducting a tracer test for denitrification beds • These data are valuable to researchers investigating the hydraulic performance of denitrification beds

## 1 Data

**Fig. 1:**
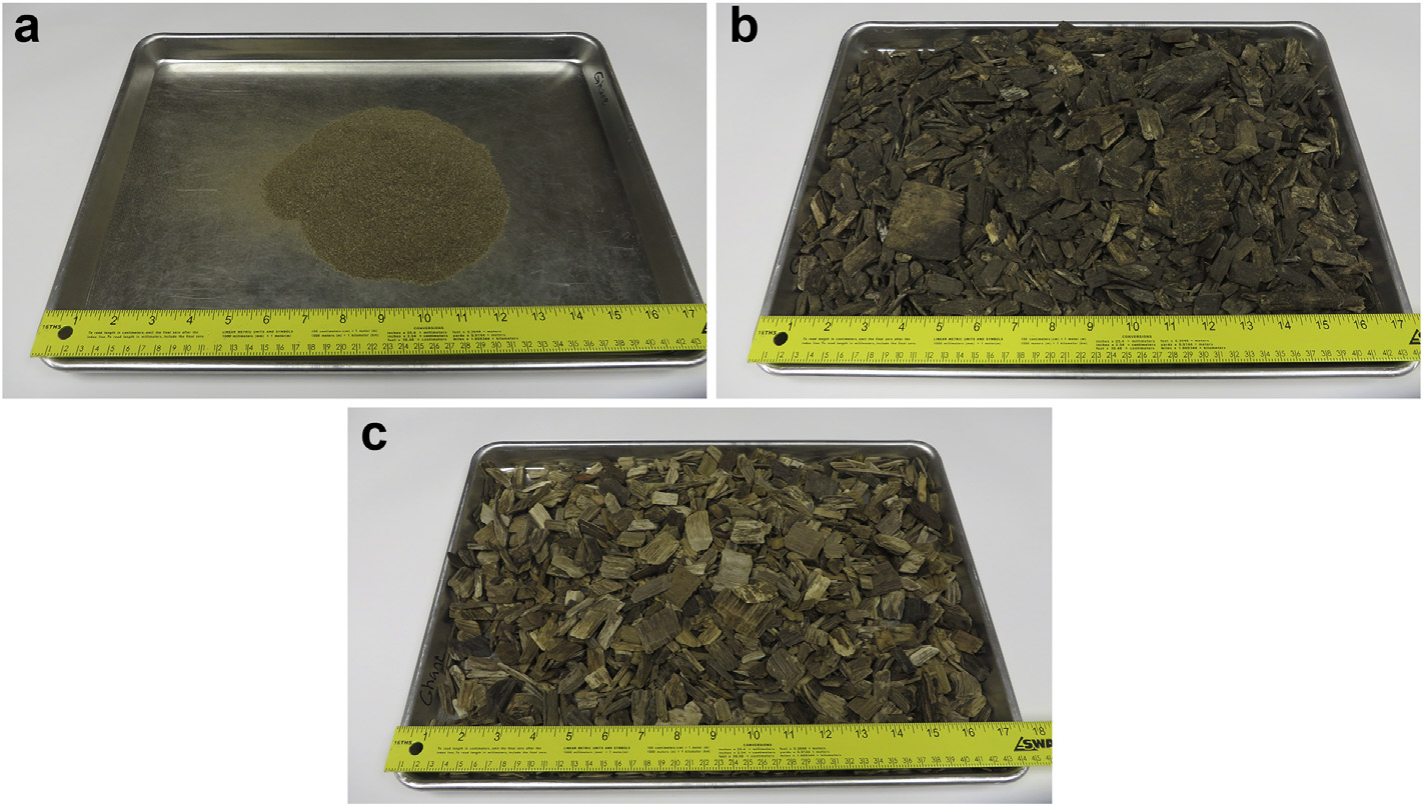
Photo of the (a) ground woodchips, (b) unwashed woodchips, and (c) washed woodchips.

**Fig. 2:**
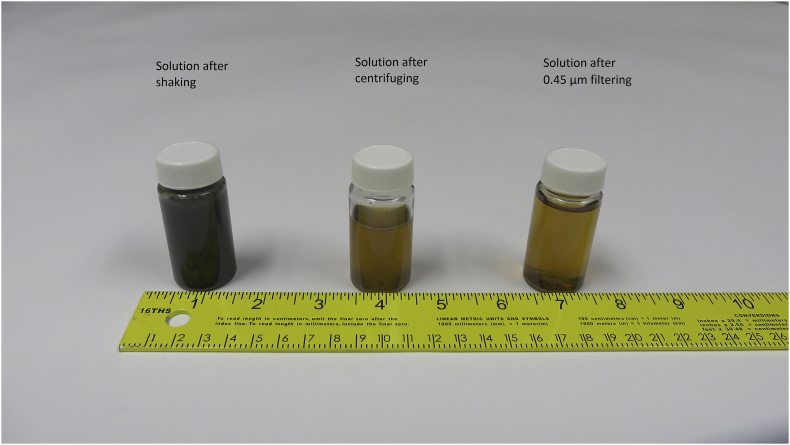
Difference between the solutions that were in contact with wood.

For the three laboratory sorption experiments, data of wood mass, drainage water volume, initial solution pH and equilibrium solution pH are presented in Table 1. Initial and equilibrium bromide concentrations as well as bromide concentration reduction after being in contact with wood are presented in Table 2. Photos of the woodchips used in the sorption experiments are shown in Fig. 1, and the solutions used for bromide concentrations are shown in Fig. 2.

**Table 1:** Summary of the bromide sorption experiments 1 (ground woodchips), 2 (unwashed woodchips), and 3 (washed woodchips).

Experiment		Sample number	Wood mass, $m_{wc}$ (kg)	Drainage water volume, $V_{w}$ (ml)	Initial solution pH	Equilibrium solution pH
Experiment 1		1	67.72 × 10^−3^	376	7.73	7.14
		2	68.29 × 10^−3^	375	7.74	6.95
		3	66.33 × 10^−3^	376	7.67	6.95
		4	67.04 × 10^−3^	375	7.67	6.95
		5	66.06 × 10^−3^	376	7.65	7.09
		6	66.27 × 10^−3^	380	7.74	6.98
		Control	66.41 × 10^−3^	376	7.72	7.03
Experiment 2		1	61.96 × 10^−3^	191	8.17	7.03
		2	62.82 × 10^−3^	190	8.19	6.91
		3	63.70 × 10^−3^	195	8.18	6.94
		4	62.11 × 10^−3^	190	8.16	6.84
		5	61.17 × 10^−3^	190	8.15	6.90
		6	61.37 × 10^−3^	193	8.16	6.93
		Control	61.53 × 10^−3^	190	8.17	7.01
Experiment 3		1	51.41 × 10^−3^	151	8.17	6.93
		2	52.72 × 10^−3^	150	8.19	7.07
		3	53.09 × 10^−3^	154	8.18	7.04
		4	53.61 × 10^−3^	155	8.16	7.03
		5	55.01 × 10^−3^	161	8.15	6.98
		6	52.50 × 10^−3^	152	8.16	7.09
		Control	53.64 × 10^−3^	154	8.17	7.02

**Table 2:** Initial and equilibrium bromide concentrations, and bromide concentration reduced after being in contact with wood for the three sorption experiments 1 (ground woodchips), 2 (unwashed woodchips), and 3 (washed woodchips).

Experiment	Sample number	Initial bromide concentration, $C_{i}$ (mg L^−1^)	Equilibrium bromide concentration, $C_{e}$ (mg L^−1^)	Bromide concentration reduction (mg L^−1^)
Experiment 1	1	9.4	9.4	0.1
	2	21.5	21.2	0.3
	3	34.4	34.0	0.4
	4	52.9	53.4	−0.5
	5	67.6	66.6	1.0
	6	76.0	75.6	0.4
	Control	0.0	0.0	
Experiment 2	1	6.1	5.4	0.7
	2	19.5	18.4	1.1
	3	30.6	30.3	0.3
	4	44.7	45.6	−0.9
	5	57.5	58.6	−1.1
	6	69.6	71.1	−1.5
	Control	0.0	0.0	
Experiment 3	1	6.1	6.5	−0.4
	2	19.5	20.6	−1.1
	3	30.6	32.9	−2.3
	4	44.7	47.3	−2.6
	5	57.5	60.0	−2.5
	6	69.6	73.1	−3.5
	Control	0.0	0.0

**Fig. 3:**
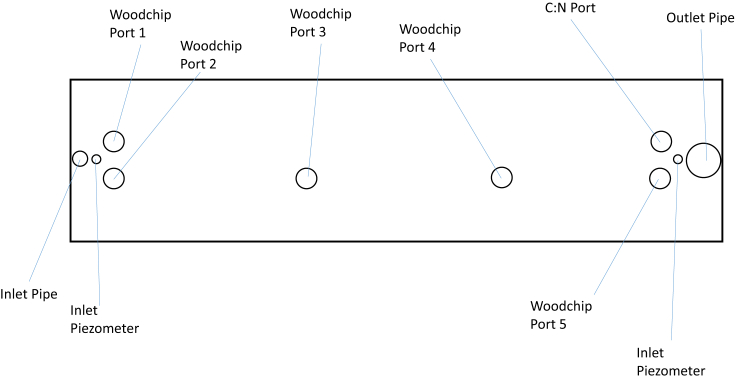
Plan view of the denitrification beds located near Willmar, Minnesota, USA. Inlet Pipe is 100-mm PVC, and the Outlet Pipe is 350-mm PVC. Woodchip Ports are 150-mm PVC, and the Inlet and Outlet Piezometers are 50-mm PVC.

**Fig. 4:**
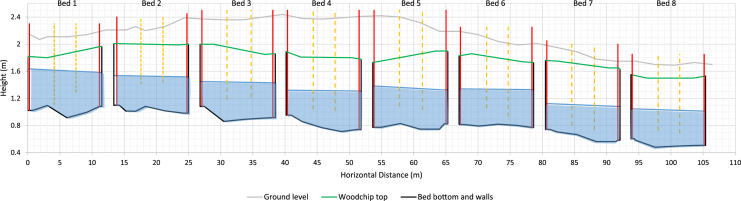
Profile of the denitrification beds 1 to 8 with water flowing from left to right in each bed. The blue shaded area is the saturated woodchip volume during the tracer tests. The solid vertical red lines are the Inlet and Outlet Pipes from left to right, and the dotted vertical orange lines are the woodchip ports 3 and 4, respectively.

**Fig. 5:**
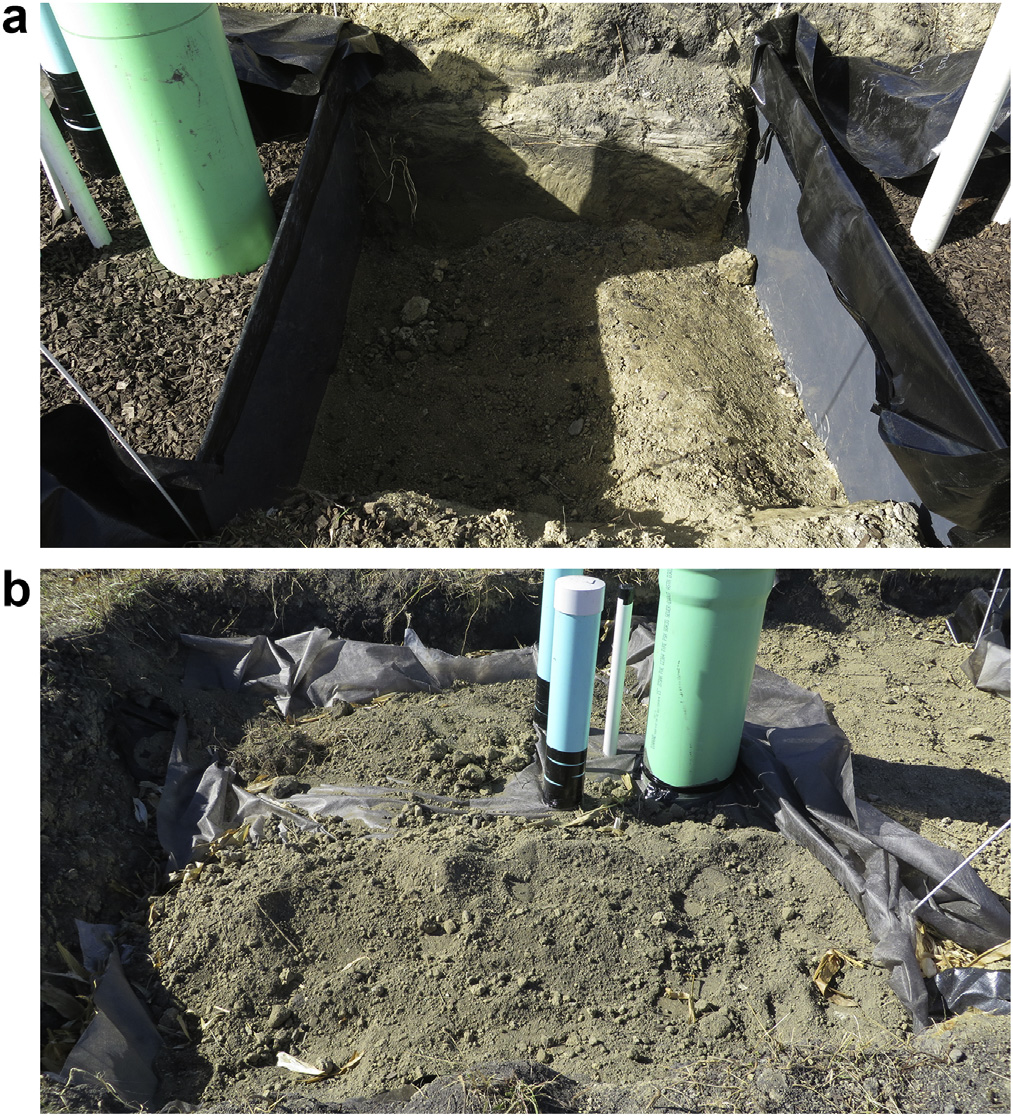
(a) Photo of the plastic sheet and soil berm, and (b) geotextile fabric to cover the denitrification bed.

**Fig. 6:**
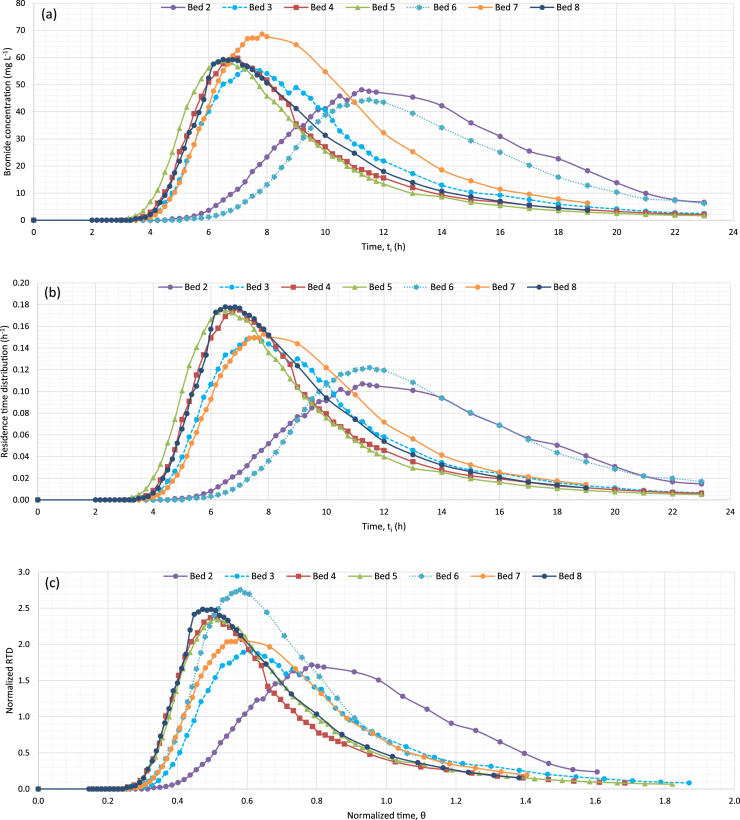
(a) Plot of bromide concentration versus time, (b) plot of residence time distribution (RTD) versus time, (c) plot of normalized RTD versus normalized time for beds 2 to 8.

For the field tracer testing, the plan view of the pipe layout in each bed, side view of the beds, method of separating the beds and covering the beds are shown in Fig. 3, Fig. 4, and Fig. 5. The data of bromide concentration versus time, residence time distribution (RTD) versus time, and normalized RTD versus normalized time are presented in Fig. 6. The data for bromide concentration over time are presented in Table 3. The theoretical retention time, volumetric efficiency and effective porosity data based on using the outflow rate compared to the average of the inflow and outflow rates are presented in Table 4 and Table 5.

**Table 3:** Bromide concentration from the tracer testing of each bed. Bromide was poured into the Inlet Pipe of each bed at time zero.

Time (h)	Br Bed 2 (mg L^−1^)	Br Bed 3 (mg L^−1^)	Br Bed 4 (mg L^−1^)	Br Bed 5 (mg L^−1^)	Br Bed 6 (mg L^−1^)	Br Bed 7 (mg L^−1^)	Br Bed 8 (mg L^−1^)
0.00	0.0	0.0	0.0	0.0	0.0	0.0	0.0
3.00	0.0	0.0	0.0	0.0	0.0	0.0	0.0
3.25	0.0	0.0	0.0	0.8	0.0	0.0	0.0
3.50	0.0	0.5	0.7	1.7	0.0	0.0	0.0
3.75	0.0	0.8	1.3	3.4	0.0	0.0	0.0
4.00	0.0	1.6	2.9	6.9	0.0	0.0	0.0
4.25	0.0	3.4	6.3	11.1	0.0	0.0	0.0
4.50	0.0	6.4	10.4	17.7	0.0	0.0	0.0
4.75	0.5	9.9	15.8	25.0	0.0	0.0	0.0
5.00	0.6	14.9	25.3	33.9	0.0	0.0	0.0
5.25	1.0	21.8	31.0	41.8	0.5	0.0	0.5
5.50	1.5	27.8	39.3	47.4	0.6	0.5	0.7
5.75	2.5	35.6	45.6	52.1	0.8	0.7	1.0
6.00	3.6	40.1	50.9	56.1	1.2	1.1	2.1
6.25	5.4	45.3	54.0	58.3	1.7	2.0	3.6
6.50	7.4	50.2	57.7	58.8	2.5	3.2	6.6
6.75	9.5	51.2	59.2	58.1	3.5	5.1	9.2
7.00	11.4	53.7	59.7	56.6	4.9	7.7	12.5
7.25	14.1	55.6	57.0	55.8	6.8	10.3	17.4
7.50	17.9	56.1	55.9	53.0	8.9	13.8	21.8
7.75	20.5	55.1	53.7	49.3	10.5	18.1	26.5
8.00	23.3	54.1	51.7	45.8	13.1	23.5	32.3
8.25	26.6	52.2	48.2	43.7	15.9	27.9	34.9
8.50	29.0	50.3	45.2	40.9	19.4	33.6	39.7
8.75	31.7	46.9	42.7	37.6	22.7	37.3	44.5
9.00	34.4	48.9	35.5	35.0	26.7	41.7	52.4
9.25	34.9	46.8	33.1	32.0	30.4	47.6	57.6
9.50	38.1	44.9	31.0	30.0	33.9	51.4	58.4
9.75	40.7	41.5	28.6	27.7	36.3	55.1	59.2
10.00	41.1	40.6	27.1	25.5	38.7	57.5	58.9
10.25	43.5	36.7	24.5	23.6	40.1	60.7	59.2
10.50	45.8	32.9	23.0	22.6	42.1	62.7	58.8
10.75	44.2	30.7	21.6	19.9	42.5	64.7	57.2
11.00	46.6	28.1	19.4	18.5	43.6	66.9	56.6
11.25	48.1	27.1	18.7	17.0	44.0	67.2	55.6
11.50	47.6	24.7	17.5	15.5	44.4	67.1	53.5
11.75	47.2	22.7	16.4	14.4	43.7	68.6	52.4
12.00	NA	21.9	15.6	13.4	43.5	67.7	50.6
13.00	45.4	17.2	12.0	9.9	39.4	64.7	41.2
14.00	42.2	12.9	9.3	8.6	34.1	54.7	31.3
15.00	35.9	10.4	7.6	6.6	29.3	43.5	24.7
16.00	30.9	9.2	6.6	5.4	25.0	32.2	18.0
17.00	25.5	7.6	5.5	4.3	20.2	25.2	13.9
18.00	22.6	6.0	4.4	3.5	15.8	18.6	10.7
19.00	18.2	4.9	3.8	3.0	12.8	14.5	8.7
20.00	13.8	4.1	3.2	2.5	10.3	11.4	7.0
21.00	9.9	3.3	2.7	2.1	7.9	9.6	5.5
22.00	7.5	2.8	2.3	1.8	7.2	7.8	4.5
23.00	6.6	2.5	2.1	1.7	6.2	6.3	3.7

**Table 4:** Theoretical retention time for beds 2 to 8 when using the outflow rate compared to the average of the inflow and outflow rates, and the overestimation error associated with using outflow rate.

Bed number	Water recovery fraction, R	Theoretical retention time based on outflow rate (h)	Theoretical retention time based on average flow rate, $t_{n}$ (h)	Overestimation when using outflow rate (%)
2	0.81	16.04	14.32	12.0
3	0.93	12.77	12.30	3.8
4	1.00	13.65	13.65	0.0
5	0.89	13.42	12.62	6.3
6	0.78	22.59	19.81	14.0
7	0.98	13.64	13.54	0.7
8	0.97	13.97	13.76	1.5

**Table 5:** Volumetric efficiency and effective porosity of beds 2 to 8 when using the outflow rate and the average of the inflow and outlfow rates.

Bed number	Volumetric efficiency based on outflow rate	Volumetric efficiency based on average flow rate	Effective porosity based on outflow rate	Effective porosity based on average flow rate
2	0.84	0.95	0.72	0.80
3	0.78	0.81	0.66	0.69
4	0.68	0.68	0.58	0.58
5	0.66	0.70	0.56	0.60
6	0.60	0.69	0.51	0.58
7	0.73	0.74	0.62	0.63
8	0.66	0.67	0.56	0.57

## 2 Experimental design, materials, and methods

### 2.1 Bromide sorption experiments

For sorption experiment 1, air-dried woodchips were ground into particle size of <1 mm (Fig. 1). For sorption experiments 2 and 3, unwashed and washed woodchips were used, respectively (Fig. 1). A known mass of wood was placed in contact with drainage water with a known concentration of bromide and was shaken for 5 hours for experiment 1 and was shaken for 1 hour for experiments 2 and 3. After the shaking process, the solutions were centrifuged, and the solutions were collected by filtering through a 0.45-μm sterile syringe filter (VWR, Radnor, Pennsylvania, USA). Fig. 2 shows the difference between the solution after shaking, after centrifuging, and after filtering. Filtered solutions were analyzed for bromide concentration.

To determine the initial- and filtered-solution concentrations of bromide, the solutions were analyzed within 11 days by colorimetry (Lachat QuikChem 8500 Flow Injection Analysis, Hach Co., Loveland, CO, USA) based on the QuikChem method 10-135-21-2-B. We made standard bromide concentrations using the yellow-colored drainage water that had been in contact with woodchips to check the concentration data. After checking the data, we found that the yellow color of the filtered solutions interfered with the measurements (Fig. 2), and caused the colorimetric method to underestimate the bromide concentrations. Therefore, we used ion chromatography (Thermo Scientific, Dionex Integrion HPIC, San Jose, CA, USA) to determine the bromide concentration within 44 days.

### 2.2 Bromide tracer experiments

A known mass of potassium bromide (400 g of KBr) was dissolved in water. We poured the tracer solution into the inlet pipe (Fig. 3) of the seven denitrification beds (Fig. 4) in less than 30 seconds. The beds were separated from one another with a plastic sheet and soil berm (Fig. 5a), and they were covered with a geotextile fabric (Fig. 5b). Automated samplers were setup at the outlet to collect water samples from the outflow beginning with more frequent samples during the rising limb of the hydrograph, and less frequent sampling during the falling limb of the hydrograph. Determining when to increase and decrease sampling frequency was estimated from a preliminary tracer testing that is not published here.

Once water samples were analyzed for bromide concentration, tracer concentration versus time was plotted (Fig. 6a). An important check to determine if the peak concentration has been included in the sampling is to look for the presence of a short plateau, consisting of two or more points with similar concentrations that are close in time (Fig. 6a). If there is a sharp peak, the peak concentration may have been missed. Capturing the peak will be important when calculating the hydraulic index that relies on the time of the peak. Furthermore, the variable frequency of water sampling at the outlet pipes provided a high-resolution curve. Water samples from the tracer tests were analyzed for bromide within 6 weeks by colorimetry (Lachat QuikChem 8500 Flow Injection Analysis, Hach Co., Loveland, CO, USA) based on the QuikChem method 10-135-21-2-B. Table 3 shows the bromide concentrations over time for the tracer testing of each denitrification bed. The plot of temporally normalized RTD versus normalized time [2] was used to compare between denitrification beds (Fig. 6c). Interpretation of the data can be found in the related research article [1].

The average of the inflow and outflow rates (L min^−1^) of the bed ($Q_{ave}$) was used in the calculation of the nominal (theoretical) retention time as [3]

(1) $$t_{n}=\frac{V_{s}n}{60Q_{ave}}$$

where $V_{s}$ is the saturated volume of the bed (L), n is total porosity of woodchips. If the water recovery fraction (R=outflow/inflow) is 0.5 < R < 2.0, the approximation of $Q_{ave}$ will provide the nominal retention time with 4% accuracy [3]. A total porosity of 0.85 from Ghane et al. (2014) [4] was used to calculate the theoretical retention time.

When a denitrification bed is waterproof, either of the inflow or outflow rate can be used in Eq. (1) because inflow is equal to outflow. However, using the average of the inflow and outflow rate becomes important when the denitrification bed leaks or allows water to seep into it. Data in Table 4 show that $t_{n}$ is overestimated using the outflow rate when a denitrification bed leaks. Only bed number 4 did not provide overestimation due to equal inflow and outflow rates. When using the outflow rate in a leaky system, volumetric efficiency and effective porosity will be underestimated (Table 5).
