# Supplementary material for: Data of bromide sorption experiments with woodchips and tracer testing of denitrification beds
Source: Data Brief. 2019 Apr 17;24:103914. doi: 10.1016/j.dib.2019.103914 (PMC6502746; doi:10.1016/j.dib.2019.103914)
Supplement: Multimedia component 1 [file mmc1.docx]

**Declaration of interests**

The authors declare that they have no known competing financial interests or personal relationships that could have appeared to influence the work reported in this paper.

The authors declare the following financial interests/personal relationships which may be considered as potential competing interests:
